# Short‐term effect of dressing with Dermaheal ointment in the treatment of diabetic foot ulcer: A double‐blinded randomized controlled clinical trial

**DOI:** 10.1002/hsr2.1868

**Published:** 2024-02-13

**Authors:** Pouya Salahi, Morteza Nasiri, Leila Yazdanpanah, Sepehr Khosravi, Mohammad Reza Amini

**Affiliations:** ^1^ Endocrinology and Metabolism Research Center, Endocrinology and Metabolism Clinical Sciences Institute Tehran University of Medical Sciences Tehran Iran; ^2^ Department of Operating Room Nursing, School of Allied Medical Sciences Tehran University of Medical Sciences Tehran Iran; ^3^ Health Research Institute, Diabetes Research Center Ahvaz Jundishapur University of Medical Sciences Ahvaz Iran; ^4^ Non‐Communicable Diseases Research Center, Endocrinology and Metabolism Population Sciences Institute Tehran University of Medical Sciences Tehran Iran; ^5^ Diabetes Research Center, Endocrinology and Metabolism Clinical Sciences Institute Tehran University of Medical Sciences Tehran Iran

**Keywords:** diabetic foot, herbal medicine, skin ulcer, wound healing

## Abstract

**Background and Aims:**

Diabetic foot ulcers, a major cause of amputations in diabetics, could benefit from natural products as adjuncts to standard care, given the costs and adverse effects of typical therapies. This study aims to evaluate the short‐term effects of dressing with Dermaheal ointment in the treatment of DFUs through a double‐blinded randomized controlled clinical trial.

**Methods:**

This double‐blinded, placebo‐controlled trial included 50 patients with Wagner's ulcer grade I or II, randomly assigned to Dermaheal and placebo groups (received standard treatment and placebo ointment). The ulcer site was dressed daily for four consecutive weeks with either Dermaheal or placebo ointment. Ulcer healing score (using DFU healing checklist), ulcer size with transparent ruler and largest dimension of ulcer, and pain severity using numerical pain rating score (were recorded at five‐time points, including baseline, and on weeks 1, 2, 3, and 4). Also, ulcer healing status was investigated at the trial ended in November 2021.

**Results:**

Both groups showed significant improvement in ulcer healing over 4 weeks (*p*_time_ < 0.001), with more remarkable progress in the Dermaheal group (*p*_group_ = 0.03). At the trial end, complete ulcer healing was also significantly higher in the Dermaheal group compared to the placebo group (56% vs. 12%, *p* = 0.002). Both groups exhibited a decrease in ulcer size (*p*_time_ < 0.001). Considering the baseline ulcer size as a covariate, substantial changes in mean ulcer size were noted in the initial (*p* = 0.01), second (*p* = 0.001), third (*p* = 0.002), and fourth (*p* = 0.002) weeks of the intervention, showing a preference for the Dermaheal group. However, no significant between‐group difference was observed in pain severity levels.

**Conclusion:**

Dressing with Dermaheal as a topical treatment shows promise in improving healing and reducing the size of diabetic foot ulcers. Further research is needed to confirm these findings' long‐term efficacy.

## Key messages

- Ulcer healing significantly improved in the Dermaheal group compared to the placebo group.
- Reduction in ulcer size was significantly more in the Dermaheal group than in the placebo group.
- The pain‐alleviating effect of Dermaheal was not superior to the placebo.

## 1 INTRODUCTION

Diabetes mellitus (DM) and its associated complications are major contributors to premature mortality in many countries.^1^ Diabetic Foot Ulcers (DFUs) have a global prevalence of 6.3% (95% confidence interval [CI]: 5.4%–7.3%) and are the main reason for hospitalizations in patients with diabetes.^2^ Furthermore, it is the most prevalent cause of nontraumatic amputations. Many studies propose that up to 85% of amputations in patients with DM can be prevented with sufficient control of risk factors and proper and proven treatments.^2^

The basic standards of DFU care include debridement approaches, daily dressings, offloading techniques, and systemic antibiotic therapies, which are costly or need regular admission to health centers.^3^ The continuous search for cost‐effective alternatives has led to daily exploration of innovative adjunctive therapies, including topical cellular and acellular growth factors, oxygen therapies, negative pressure wound therapy, shock wave therapy, and modern dressings.^4^, ^5^ Nevertheless, no high‐quality evidence supports the usage of these therapies without problem, resulting in an ongoing challenge in managing the DFU.^6^ Topical treatments, particularly agent‐based dressings, offer simplicity in application compared to other adjunctive methods, with their availability increasing tenfold in the last decade^7^ In recent review studies, the DFU healing rate of the ointments varied from 61% to 63.6%.^8^ Moreover, systematic reviews emphasize the potential of topical and natural herbal agents as cost‐effective, adjunctive nonpharmacological measures, demonstrating favorable outcomes with minimal adverse reactions in DFU healing.^9^, ^10^ The bioactive molecules derived from therapeutic herbs are believed to exert multiple pharmacological effects, contributing to DFU control with minimal adverse consequences.^11^ Dermaheal, an ointment comprising therapeutic herbs, including those studied in diabetic rats, has shown promising results in healing cutaneous wounds. In a study involving 36 male rats, two experimental groups received either olive leaf ointment or Dermaheal ointment, with diabetes induced by a streptozotocin injection. Notably, the group receiving Dermaheal ointment demonstrated significantly higher wound contraction compared to the group treated with olive leaf ointment. These findings highlight the potential of Dermaheal in enhancing both the macroscopic and microscopic aspects of wound healing in diabetic conditions.^12^

Thus, this study aims to evaluate the short‐term therapeutic effects of dressing with Dermaheal ointment in the treatment of DFUs through a double‐blinded randomized controlled clinical trial. To this end, we compared the effectiveness of daily dressing with topical administration of Dermaheal and placebo ointments on DFU healing, size, and pain severity.

## 2 METHODS

### 2.1 Study design

This is a randomized, placebo‐controlled, double‐blinded, parallel‐group clinical trial. The study protocol was registered in the Iranian Registry of Clinical Trials (No. IRCT20080904001199N4). The study was also reported based on Consolidated Standards of Reporting Trials (CONSORT) statements for presenting parallel‐group randomized trials and herbal interventions.^13^ Also, we followed the guidelines for reporting statistics for clinical research in urology.^14^

### 2.2 Ethical considerations

The Institutional Review Board and Ethics Committee of the Endocrine and Metabolism Research Institute (EMRI), Tehran University of Medical Sciences (TUMS), Tehran, Iran, granted approval for this study (No. IR.TUMS.EMRI.REC.1399.036). All procedures adhered to the principles of the Declaration of Helsinki, and written informed consent was acquired from all patients before their enrollment.

### 2.3 Study setting and participants

Individuals with type 1 and 2 diabetes who were receiving routine care at the research institute of the corresponding author between February 2019 and September 2021 were invited to take part in this study. The inclusion criteria were as follows: (1) being in the age range of 18–80 years, (2) with a body mass index (BMI) ranging from 18 to 30 kg/m², (3) having an ankle‐brachial pressure index (a noninvasive test to assess severity of peripheral arterial disease in DFUs) of 0.7–1.2, and (4) having Wagner's grade (used to classify the severity of DFUs on a scale of 0 to 5) I or II DFU for more than 4 weeks in one or more areas of the low‐pressure points of the feet (e.g., toes, soles, heels, dorsum).^15^ The exclusion criteria were as follows: (1) presence of any fracture and trauma on the affected foot and evidence of osteomyelitis; (2) having foot gangrene requiring amputation; (3) requiring intravenous antibiotic therapy; (4) having taken medications in the previous 3 months that could potentially interact with the therapeutic study protocol, thereby impeding the ulcer healing process (such as glucocorticoids, anticoagulants, immunosuppressive, and cytotoxic agents),^16^ (5) having a history of comorbidities that may affect the ulcer healing (e.g., cancers, vasculitis, advanced heart failures, renal and liver failures, rheumatoid arthritis, coagulopathy disorders), (6) having a history of substance abuse or opioid/alcohol addiction, (7) consumption of other herbal medicines including topical or oral forms in the most recent 3 months,^17^ (8) having any allergic reactions to the herbal extracts, (9) having a history of participating in any foot preventive educational programs.

Also, In this study, participants who missed more than two treatment sessions during the 4‐week trial period were considered dropouts.

### 2.4 Sample size

Based on data obtained for DFU healing from a recent study,^16^ a total sample size of 21 patients in each group was estimated, bearing in mind the following sample size formula recommended for two‐arm randomized clinical trials and type I error of 1% (*α* = 0.01) and type II error of 20% (*β* = 0.20). Taking into account the potential occurrence of a 15% sample dropout, 25 participants were chosen for each study group.

n=z1−α2+z1−β2(S12+S22)(μ¯1−μ¯2)2=(2.576+0.84)2  [(29.84)2+(43.91)2](365−325)2=21.

### 2.5 Sampling and randomization

Patients meeting eligibility criteria were chosen through a consecutive sampling method. Utilizing the shuffling sealed envelopes technique, they were randomly assigned to either the Dermaheal group (*n* = 25) or the placebo group (*n* = 25). In this process, 50 codes (25 codes labeled A for Dermaheal and 25 codes labeled B for placebo) were inscribed on individual sheets and enclosed within sealed envelopes. While undergoing the admission process, patients made a random selection of an envelope, and subsequently, their allocation to either the Dermaheal or placebo group was determined by the code contained within the chosen envelope. The first investigator, who was the only person who knew what the allocation codes meant, handled the sampling and randomization processes for the study.

### 2.6 Blinding

The Dermaheal and placebo were prepared and coded by the first investigator in tubes of identical appearance. The patients, along with the initial nursing assistant responsible for applying the topical products, were kept unaware of the contents of the tubes. Furthermore, the assistant in charge of gathering data on DFUs and the second nursing assistant overseeing standard care remained uninformed about the group assignments.

### 2.7 Data collection

#### 2.7.1 Demographic and clinical data

Demographic and clinical information was collected using a researcher‐designed questionnaire. This questionnaire encompassed details on quantitative variables, such as age, BMI, duration of diabetes and foot ulcer, fasting blood sugar (FBS), glycosylated hemoglobin (HbA1C), systolic and diastolic blood pressures, and ankle–brachial pressure index. It also included qualitative variables like gender, marital status, educational level, occupation, diabetes type, history of other diseases, diabetes complications, amputation, and hospitalization, type of diabetes treatment, ulcer location, ulcer grade based on the Wagner's system,^18^ presence of foot edema and infection, presence of foot and nail deformities, and palpation status of dorsalis pedis and posterior tibial pulses. FBS and HbA1C values were extracted from the patients' latest laboratory reports conducted 1–2 weeks before the study initiation. The authors of this study performed both the measurement of height and weight, as well as the calculation of BMI. BMI was calculated by dividing the weight in kilograms by the square of the height in meters, and these measurements were conducted by the authors themselves. In the study, height and weight measurements are undertaken using specific methods to ensure accuracy, and these measurements are crucial for the calculation of BMI. For height measurement, the procedure involves using a meter and a ruler. Weight measurement is conducted using calibrated digital scales, specifically Innofit scales. The calibration process is critical to ensure the accuracy of these measurements and was conducted regularly.

Also, as the data collection of this study happened during COVID‐19 pandemic and in response to this pandemic, we implemented targeted measures to protect the physical and psychological well‐being of our participants, especially considering their increased risk due to diabetes. To minimize COVID‐19 exposure, we provided personal driver services for safe transportation to and from the clinic, significantly reducing the risk associated with public transport and crowded spaces. In the clinic, we staggered appointment times to maintain social distancing and prevent crowding. These steps not only ensured participant safety but also likely reduced pandemic‐related anxiety, contributing to the psychological comfort of our participants. By maintaining the continuity of care and adapting our procedures to the pandemic conditions, we were able to preserve the integrity of our data collection and uphold the reliability of our study during this challenging period.

#### 2.7.2 DFU healing

The DFU healing checklist was used to record the score and status of DFU healing as the primary outcome (Supporting Information S1: Material 1). Based on this tool, four ulcer parameters were recorded at the end of each week, including ulcer degree (i.e., score and stage), ulcer color (i.e., center and periphery), ulcer peripheral tissues (i.e., color, hotness, edema, sense), and ulcer exudates (i.e., color, odor, amount). Each parameter obtained a maximum score of 100. The overall score spans from 50 to 400, where higher scores signify improved healing of DFUs. Based on the total scores recorded on the last week and the first week, the ulcer healing status is classified into four conditions as follows: (1) complete healing, obtaining a score of 400 on the last week; (2) partial healing, increasing a minimum 30 scores on the last week compared to the first week; (3) non‐healing is defined as the absence of any alterations in the last‐week and first‐week scores or a change in the score of less than 30 points; and (4) deterioration of ulcer: decreasing of a minimum of 10 scores on the last week compared to the first week.^16^ The DFU healing checklist has been used in different studies as a valid measure.^19^, ^20^, ^21^ Nasiri et al. (2015) confirmed the content validity of this checklist.^16^ Also, Mahmodi^22^ and Sadeghi Moghadam^23^ proved the internal stability of this checklist for the first time through Cronbach's *α* of 0.90 and 0.95, respectively.^22^, ^23^ In this study, the inter‐rater reliability of the checklist was assessed by two trained nurses possessing similar professional characteristics. The interclass correlation coefficient assessing agreement between the raters was determined to be 0.94 for the total score and ranged from 0.81 to 0.92 for the ulcer parameters.

#### 2.7.3 DFU size

For the measurement of ulcer size, a secondary outcome, we utilized a transparent, scaled, flexible ruler to record the largest dimensions of the ulcer site. Subsequently, the ulcer size was calculated by multiplying the largest length by the largest width and the largest depth.^24^

#### 2.7.4 DFU‐induced pain severity

We employed a 0–10 Numerical Pain Rating Scale (NPRS) to assess pain severity with regard to the DFU location, a secondary outcome. Patients expressed the severity of their pain by selecting a point on a continuous line with endpoints spanning from 0 to 10, where 0 denotes “not at all experienced” and 10 signifies “highly experienced”). The NPRS is a straightforward instrument, known for its ease of administration, and has demonstrated reliability and validity in various studies for assessing pain intensity associated with DFUs.^25^, ^26^ Building on a prior analysis, the total score of the NPRS was classified into categories: none (score: 0, 1), mild (score: 2, 3), moderate (score: 4, 5), severe (score: 6), and very severe (score: 7–10).^27^

#### 2.7.5 Adverse effects of the intervention

As one of the secondary outcomes, the incidence of all adverse effects was documented in a form presented in a previous trial.^16^ During daily visits, the DFU specialist carefully examined all recruited patients to detect any potential adverse effects of the interventions (e.g., ulcer site sensitivity, bleeding, and infection). Also, all patients were invited to report the adverse effects they experienced.

### 2.8 Intervention

First, the patient's ulcer was assessed by a blinded DFU specialist in a private room of the recruitment center. Patients with grade I or II in Wagner's system were assessed for additional inclusion criteria. Following that, demographic and clinical information were collected for every eligible patient through interviews and the examination of clinical records. Consequently, the study outcomes, including ulcer healing score, ulcer size, and ulcer‐induced pain severity, were rated through interviews and recorded as the baseline data.

Both groups of patients received standard care; however, in the Dermaheal group, the ulcer was treated with Dermaheal ointment, whereas in the placebo group, the ulcer was treated with a placebo. The study interventions and standard care were implemented in the exact center from the first day of patients' recruitment and continued daily for four consecutive weeks. To this end, all patients were requested to refer to the recruitment center at the appointed time. The Dermaheal group was scheduled to visit the center on the morning shift (from 8:00 a.m. to 2:00 p.m.). In contrast, to avoid communication between the study groups, the placebo group was expected to visit on the evening shift (from 2:00 p.m. to 8:00 p.m.). Before each scheduled visit, the patients received reminders through phone calls.

The same standard care protocol for DFUs was implemented in the two groups by the second nursing assistant under the supervision of the assistant DFU specialist. All patients received oral antibiotics (i.e., clindamycin 600 mg every 8 h or/and ciprofloxacin 500 mg every 12 h for a minimum of 2 weeks) and local sharp debridement at any time required during the trial period. For the offloading, each individual was offered either custom‐made insole or footwear, and a decision was made at the individual level for each patient. Additionally, the periphery and center of the ulcer site underwent daily irrigation and cleansing with 1000 mL of normal saline solution (0.9%), followed by dressing with sterile gauze if necessary. Furthermore, on the initial day of recruitment, all patients received a 45‐min in‐person instruction from the nursing assistant regarding how to adhere to the standard care protocol for DFU and how to report study‐related outcomes. Additionally, patients were advised to strictly follow their prescribed medications and refrain from altering their ulcer dressings outside of the interventions provided by the study. After the instructional session, every patient was provided with a compact disk containing the session's instructions for further review. Moreover, the patient's adherence to standard care was checked during daily visits, and any issues were resolved.

To implement the study interventions, patients in both groups comfortably lay in an electric bed, and their legs were supported on the bed. Afterward, the periphery and center of the ulcer site were cleansed with 1000 mL of sterile normal saline solution (0.9%). Following this, the area was dried using sterile gauze. Following the drying process, Dermaheal or placebo ointment was administered to the ulcer, spreading from the surface to the depth using an abaisse‐langue, with the application thickness approximately 1 mm, based on the ulcer's area and depth. The ulcer site was promptly dressed with a four‐layer sterile dry gauze and secured with hypoallergenic adhesive. This procedure was carried out once a day for 4 weeks, resulting in a total intervention time of 420 min for each patient (allowing for 15 min per visit). The initial nursing assistant conducted the interventions in a private room at the recruitment center for both groups. To mitigate bias, the nursing assistant received instructions on intervention application during a practical session led by the DFU specialist, who possessed expertise in dressing DFUs with herbal medicine. At the conclusion of each intervention week (i.e., the first, second, third, and fourth weeks of intervention), the DFU specialist assessed and recorded the ulcer healing score, ulcer size, and ulcer‐induced pain severity. Furthermore, the occurrence of potential adverse events was evaluated and documented during daily visits.

Derived from a prior investigation,^12^ we utilized Dermaheal ointment produced by Darudarman Salafchegan Knowledge‐Based Co. (health production license no. S‐93‐0243, production serial no. ZD‐607). The practical components of this ointment were reported as *Arnebia euchromatin*, thymes, *Maticaria chamomilla* L., *Curcuma longa*, wax, honey, and olive oil. In our study, the application of Dermaheal involved the administration of a thin layer of the ointment to cover the entire surface of the ulcer once a day, following the changing of the dressing. The placebo cream, primarily composed of Eucerin with the addition of Vaseline, was administered by Darudarman Salafchegan Knowledge‐Based Co. in Tehran, Iran. This placebo closely resembled the Dermaheal ointment in appearance and was used in the study. The placebo, designed solely for moisturization, was applied once a day following the changing of the wound dressing. The amount applied was sufficient to entirely cover the ulcer area with a thin layer, providing consistent hydration without introducing therapeutic agents or active ingredients beyond its moisturizing effect. Previous trials did not reveal any adverse effects associated with the external application of these products.^12^, ^28^ Furthermore, in a preliminary study comprising 10 patients conducted over a 1‐month period, no adverse effects were noted following the topical application of these products (results not yet published).

### 2.9 Statistical methods

The collected data were subjected to analysis using the Statistical Package for Social Sciences software (SPSS, version 26.00; SPSS Inc.), and two‐tailed *p* values ≤0.05 were deemed significant for all analyses. Independent sample *t*‐test, Mann–Whitney *U* test, and Chi‐squared test (or Fisher's exact test) were employed to evaluate the homogeneity of groups concerning demographic and baseline clinical characteristics for quantitative, ordinal, and nominal variables, respectively. Additionally, repeated measures analysis of variance (rANOVA: Greenhouse–Geisser) was utilized to assess ulcer size and the ulcer healing score at each endpoint, taking into account time, group, and time × group.^29^ Given the significant change over time indicated by this test, Bonferroni post hoc analysis was employed for multiple comparisons of endpoints. Furthermore, as rANOVA revealed no significant difference in time × group, analysis of covariance (ANCOVA) was applied to compare the mean changes during the first, second, third, and fourth weeks of the intervention compared to the baseline between the study groups, considering baseline values as covariates. Finally, the Mann–Whitney *U* test was employed to compare groups regarding various ulcer healing status categories determined at the trial's end. This test was also utilized to compare different pain severity categories between the groups.

## 3 RESULTS

### 3.1 Patients' follow‐up

Out of the 63 eligible patients, 12 did not meet the inclusion criteria, and one declined to participate. Among the remaining 50 patients, all strictly adhered to the study protocol and were consequently included in the final analysis (Supporting Information S1: Material 2).

### 3.2 Patients' characteristics

No significant differences were observed between the two groups in terms of demographic and baseline clinical data, with a specific focus on ulcer characteristics (Table 1).

**Table 1:** Demographic and clinical variables of patients with diabetic foot ulcer.

	Dermaheal group (*n* = 25)	Placebo group (*n* = 25)	
Qualitative variables	*N* (%)	*N* (%)	*p* Value
Gender			
Male	19 (76)	17 (68)	0.7^a^
Female	6 (24)	8 (32)	
Marital status			
Married	23 (92)	22 (88)	>0.99^b^
Single	2 (8)	2 (8)	
Divorced	0 (0)	1 (4)	
Occupation			
Housewife	6 (24)	7 (28)	0.8^b^
Employed	8 (32)	6 (24)	
Unemployed	0 (0)	1 (4)	
Retired	11 (44)	11 (44)	
Educational level			
Less than a diploma	18 (72)	16 (64)	0.5^c^
Diploma	5 (20)	7 (28)	
Collegiate	2 (8)	2 (8)	
Diabetes type			
Type 1	5 (20)	1 (4)	0.7^a^
Type 2	20 (80)	24 (96)	
History of other diseases			
Yes (i.e., Hypertenstion)	15 (60)	14 (56)	>0.99^a^
No	10 (40)	11 (44)	
History of diabetes complications			
Yes (i.e., retinopathy)	16 (64)	14 (56)	0.7^a^
No	9 (36)	11 (44)	
History of amputation			
Yes	1 (4)	3 (12)	0.6^a^
No	24 (96)	22 (88)	
History of hospitalization			
Yes	15 (60)	18 (72)	0.5^a^
No	10 (40)	7 (28)	
Type of diabetes treatment^d^			
Oral agents	10 (40)	10 (40)	>0.99^b^
Insulin injection	9 (36)	9 (36)	
Oral agents plus insulin injection	6 (24)	6 (24)	
Ulcer location			
Soles	8 (32)	2 (8)	>0.99^b^
Heels	2 (8)	2 (8)	
Dorsum	3 (12)	7 (28)	
Toes	9 (36)	13 (52)	
Leg (from ankle to knee)	3 (12)	1 (4)	
Ulcer grade (Wagner's system)			
Grade I	10 (40)	14 (56)	0.3^a^
Grade II	15 (60)	11 (44)	
Presence of foot edema			
Yes	8 (32)	8 (32)	>0.99^a^
No	17 (68)	17 (68)	
Presence of foot infection			
Yes	5 (20)	6 (24)	>0.99^a^
No	20 (80)	19 (76)	
Presence of foot deformity			
Yes	9 (36)	8 (32)	>0.99^a^
No	16 (64)	17 (68)	
Presence of nail deformity			
Yes	10 (40)	10 (40)	>0.99^a^
No	15 (60)	15 (60)	
Palpation of dorsalis pedis pulse			
Strong	22 (88)	20 (80)	0.4^c^
Palpable but diminished	3 (12)	4 (16)	
Non‐palpable	0 (0)	1 (4)	
Palpation of posterior tibial pulse			
Strong	23 (92)	21 (84)	0.3^c^
Palpable but diminished	2 (8)	3 (12)	
Non‐palpable	0 (0)	1 (4)	

Quantitative variables		**Mean ± SD**	**Mean ± SD**	
Age (years)		57.9 ± 9.88	55.6 ± 10.44	0.4^e^
BMI (kg/m^2^)		28.1 ± 3.65	27.7 ± 3.49	0.7^e^
Duration of diabetes (year)		11.0 ± 8.18	12.8 ± 7.28	0.4^e^
Duration of ulcer (days)		70.9 ± 89.23	60.8 ± 80.21	0.7^e^
Fasting blood sugar (mg/dL)		174.4 ± 56.58	189.6 ± 78.70	0.4^e^
Glycosylated hemoglobin (%)		8.3 ± 1.71	8.8 ± 2.47	0.3^e^
Systolic blood pressure (mmHg)		124.7 ± 17.13	126.0 ± 13.54	0.7^e^
Diastolic blood pressure (mmHg)		78.0 ± 9.89	80.2 ± 6.99	0.3^e^
Right ABI		0.9 ± 0.14	0.9 ± 0.08	0.4^e^
Left ABI		0.9 ± 0.15	0.9 ± 0.11	0.2^e^

*Note*: Qualitative variables have been expressed as number (percentage), while quantitative variables have been presented as mean ± SD.

Abbreviations: ABI, ankle/brachial index; BMI, body mass index.

^a^ Chi‐squared test.

^b^ Fisher's exact test.

^c^ Mann–Whitney U test.

^d^ Oral agents: This category includes Metformin, Sulfonylureas, and Thiazolidinediones. Insulin Injections: Primarily insulin pens (e.g., NovoRapid, Levemir, Lantus, Apidra), with Regular/NPH regimen utilized in certain cases. Oral agents plus insulin injection: Involves a regimen combining the aforementioned oral agents with insulin injections.

^e^ Independent samples *t*‐test.

### 3.3 Ulcer healing score and status

**Figure 1:**
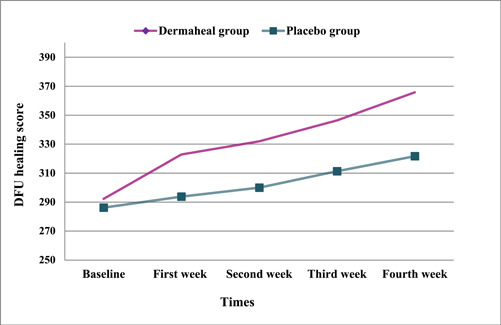
Total diabetic foot ulcer healing score of the study groups at different times.

There was a substantial increase in the total ulcer healing score of Dermaheal and placebo groups during the 4 weeks of intervention (Figure 1). Based on the rANOVA, the increase in the total ulcer healing score was significant over time (*p*_time_ < 0.001). Moreover, when employing the Bonferroni post hoc analysis for multiple comparisons, it was evident that the mean changes in the total ulcer healing score showed significant differences between most endpoints within the Dermaheal group (with a *p* value of <0.001 in the majority of cases). Also, considering only the main effect of the group, a significant difference was found in the total ulcer healing score in favor of the Dermaheal group (*p*_group_ = 0.03). However, no significant difference was revealed between groups over time (*p*_time×group_ = 0.1) (Table 2).

**Table 2:** Total ulcer healing score and ulcer size among patients with diabetic foot ulcer at different times.

Variables (times^a^)	Dermaheal group (*n* = 25)	Placebo group (*n* = 25)	Effect size	95% confidence interval	Test results^b^		
					Time	Group	Time × Group
Total ulcer healing score^c^							
Baseline	292.2 ± 52.64	286.2 ± 63.08	0.10	−27.04, 39.04	*F* = 26.8, *p* < 0.001	*F* = 4.5, *p* = 0.03	*F* = 2.1, *p* = 0.1
First week	322.8 ± 37.77^d^	293.8 ± 54.74	0.61	2.25, 55.74			
Second week	331.8 ± 41.27	300.0 ± 59.34	0.62	2.17, 61.57			
Third week	346.3 ± 42.37^d^, ^e^	311.3 ± 54.07^f^	0.72	5.44, 64.55			
Fourth week	365.7 ± 22.31^d^, ^e^, ^f^, ^g^	321.6 ± 51.12^d^, ^f^, ^g^	1.11	18.94, 69.22			
Ulcer size (cm)^h^							
Baseline	0.9 ± 1.61	1.0 ± 1.49	0.06	−0.98, 0.79	*F* = 12.8, *p* < 0.001	*F* = 1.0, *p* = 0.3	*F* = 1.1, *p* = 0.2
First week	0.4 ± 0.65	0.8 ± 1.29	0.37	−0.97, 0.19			
Second week	0.1 ± 0.21	0.6 ± 0.92	0.76	−0.89, −0.11			
Third week	0.07 ± 0.2^e^	0.3 ± 0.46	0.78	−0.49, − 0.07			
Fourth week	0.01 ± 0.03^e^, ^f^	0.2 ± 0.45	0.81	−0.46, −0.06			

*Note*: All values are reported as means ± SD.

^a^ The outcomes were recorded at five points of time, including before the intervention (baseline), and on the first, second, third, and fourth weeks of intervention.

^b^ Repeated‐measures analysis of variance (Greenhouse–Geisser).

^c^ Diabetic foot ulcer healing was measured by a four‐parameter scale: total score ranges from 50 to 400.

^d^ Significant compared to the baseline.

^e^ Significant compared to the second week.

^f^ Significant compared to the first week.

^g^ Significant compared to the third week.

^h^ Diabetic foot ulcer size was estimated by the following equation: largest length × largest width ×  largest depth.

Scores of ulcer healing parameters in each study group are presented in Supporting Information S1: Material 3. Based on the rANOVA, a significant increase was observed during the 4 weeks for all ulcer parameters (*p*_time_ < 0.001 in all cases. Nevertheless, when focusing solely on the primary effect of the group, a significant difference was observed exclusively in the ulcer degree. (*p*_group_ = 0.02). Likewise, no noteworthy difference was observed between groups over time, except for changes in ulcer color (*p*_time×group_ = 0.006).

When accounting for baseline values as covariates, the ANCOVA test revealed a significant increase in the mean changes of the total ulcer healing scores compared to the baseline during the first (*p* = 0.008), second (*p* = 0.001), third (*p* = 0.02), and fourth (*p* = 0.001) weeks of the intervention, favoring the Dermaheal group (Table 3). Such findings were also found for most ulcer parameters (Supporting Information S1: Material 4).

**Table 3:** Changes in total ulcer healing score and ulcer size among patients with diabetic foot ulcer compared to the baseline on the first, second, third, and fourth weeks of the intervention.

Variables (times^a^)	Dermaheal group (*n* = 25)	Placebo group (*n* = 25)	Effect size	95% confidence interval	Test results^b^
Total ulcer healing score^c^					
First week	31.8 ± 6.52	6.3 ± 6.52	0.78	6.80, 44.05	*F* = 7.5, *p* = 0.008
Second week	44.1 ± 8.94	15.2 ± 8.94	0.64	3.41, 54.42	*F* = 5.2, *p* = 0.02
Third week	59.1 ± 9.18	29.4 ± 9.18	0.64	3.32, 55.98	*F* = 5.1, *p* = 0.02
Fourth week	78.5 ± 8.20	37.3 ± 8.0	1.01	17.92, 64.42	*F* = 12.8, *p* = 0.001
Ulcer size (cm)^d^					
First week	−0.5 ± 0.09	−0.1 ± 0.09	0.73	−0.59, −0.07	*F* = 6.4, *p* = 0.01
Second week	−0.8 ± 0.09	−0.3 ± 0.09	1.06	−0.74, −0.21	*F* = 13.1, *p* = 0.001
Third week	−0.9 ± 0.06	−0.6 ± 0.06	0.96	−0.47, −0.11	*F* = 10.7, *p* = 0.002
Fourth week	−0.9 ± 0.06	−0.6 ± 0.06	0.96	−0.46, −0.10	*F* = 10.7, *p* = 0.002

*Note*: All values are reported as means ± standard errors.

All values are reported as means ± SD.

^a^ The outcomes were recorded at five points of time, including before the intervention (baseline), and on the first, second, third, and fourth weeks of intervention.

^b^ Analysis of covariance, considering baseline values as covariates.

^c^ Diabetic foot ulcer healing was measured by a four‐parameter scale: total score ranges from 50 to 400.

^d^ Diabetic foot ulcer size was estimated by the following equation: largest length × largest width × largest depth.

**Figure 2:**
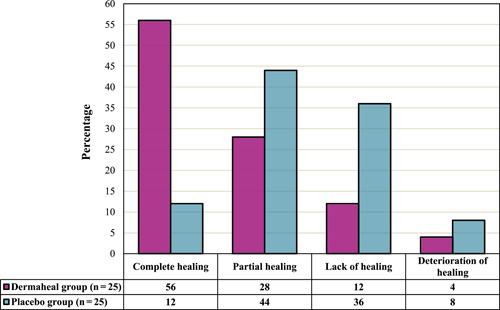
Diabetic foot ulcer healing status of the study groups at the end of the trial.

Concerning the categories of ulcer healing status at the conclusion of the 4‐week trial, a notable difference was noted between the study groups (Mann–Whitney *U* = 160.0, *p* = 0.002). As illustrated in Figure 2, complete ulcer healing was significantly more prevalent in the Dermaheal group compared to the placebo group (56% vs. 12%). The ulcer healing process in one patient from the Dermaheal group over the 4 weeks is depicted in Supporting Information S1: Material 5.

### 3.4 Ulcer size

Based on the rANOVA, a significant reduction was found in the ulcer size during the 4 weeks (*p*_time_ < 0.001). Nevertheless, considering only the primary impact of the group and taking into account the progression over time, no significant difference was identified between the groups, (*p*_group_ = 0.3, *p*_time×group_ = 0.2). As indicated by the Bonferroni post hoc analysis, there was a notable decrease in mean changes between the fourth and first weeks (*p* = 0.03), the fourth and second weeks (*p* = 0.01), and the third and second weeks (*p* = 0.01) in the Dermaheal group. Conversely, no significant intra‐group difference was observed in the placebo group (Table 2). Furthermore, when considering baseline ulcer size as a covariate, the ANCOVA test revealed a significant reduction in mean changes compared to the baseline during the first (*p* = 0.01), second (*p* = 0.001), third (*p* = 0.002), and fourth (*p* = 0.002) weeks of the intervention, favoring the Dermaheal group (Table 3).

### 3.5 Pain severity

Lack of pain was 32% at baseline in the Dermaheal group, which reached 80% at the trial end. Similarly, 28% and 56% of patients in the placebo group experienced a lack of pain at baseline and trial end, respectively. Although the pain severity was alleviated during the 4 weeks in both the Dermaheal and Placebo groups, no significant between‐group differences were found regarding pain severity categories in any endpoints (Table 4).

**Table 4:** Ulcer‐induced pain severity among patients with diabetic foot ulcer at different times.

Times^a^	Dermaheal group (*n* = 25)	Placebo group (*n* = 25)	Between‐group test results^b^
Baseline			
None	8 (32)	7 (28)	Value = 306.5, *p* = 0.9
Mild	7 (28)	7 (28)	
Moderate	6 (24)	11 (44)	
Severe	1 (4)	0 (0)	
Very severe	3 (12)	0 (1)	
First week			
None	12 (48)	9 (36)	Value = 300.5, *p* = 0.8
Mild	5 (20)	9 (36)	
Moderate	6 (24)	7 (28)	
Very severe	2 (8)	0 (0)	
Second week			
None	13 (52)	14 (56)	Value = 286.5, *p* = 0.5
Mild	5 (20)	6 (24)	
Moderate	5 (20)	5 (20)	
Severe	2 (8)	0 (0)	
Third week			
None	19 (76)	14 (56)	Value = 254.5, *p* = 0.1
Mild	3 (12)	7 (28)	
Moderate	3 (12)	4 (16)	
Fourth week			
None	20 (80)	14 (56)	Value = 243.5, *p* = 0.1
Mild	3 (12)	9 (36)	
Moderate	2 (8)	2 (8)	

*Note*: All values are reported as number (percentage).

^a^ Pain severity was measured by a 0–10 Numerical Pain Rating Scale at five points of time (i.e., baseline and on the first, second, third, and fourth weeks of interventions): the total score was classified into none (score: 0, 1), mild (score: 2, 3), moderate (score: 4, 5), severe (score: 6), and very severe (score: 7–10).

^b^ Mann–Whitney *U* test.

### 3.6 Adverse effects

Throughout the duration of the study, not even one of the patients reported encountering any adverse effects associated with the treatments.

## 4 DISCUSSION

DFUs represent a prevalent, costly, and debilitating complication among diabetic patients, with potential consequences including infection and amputation.^30^ Several approaches have been investigated to enhance treatment outcomes for this type of ulceration.^31^ For instance, recent studies have provided evidence supporting the therapeutic effectiveness of specific herbal remedies in facilitating the healing of ulcers among patients with DFU.^12^, ^32^ The findings of this study also indicate that Dermaheal ointment, formulated with multiple herbs, has the potential to contribute to the healing of diabetic DFU.

Based on the findings of the current study, ulcer healing improved and ulcer size decreased significantly in the two groups. In contrast, the improvement in ulcer healing and reduction in ulcer size was more in the Dermaheal group. Additionally, considering the baseline values as covariates, mean changes in the ulcer healing and ulcer size when compared to the initial baseline measurements were significant on all assessment weeks in patients whose ulcers were dressed with Dermaheal compared to those whose ulcers were dressed with placebo. Thus, dressing with Dermaheal ointment could improve healing and reduce ulcer size in patients with DFU. However, the pain severity did not change significantly between groups, which could be attributed to the subjective nature of pain scores compared to the objective nature of wound healing and wound size.

In the healing of DFUs, Dermaheal has demonstrated efficacy comparable to other cutting‐edge topical treatments, including topical fibrin, leukocyte platelet patch, and placenta‐derived products.^33^ In a related context, a study investigated the LeucoPatch system, which incorporates autologous leukocytes, platelets, and fibrin. The findings indicated that 34% of patients in the trial group achieved ulcer healing within 20 weeks, showcasing a quicker healing time compared to the standard care group.^34^ Likewise, in a separate study, the application of sucrose octasulfate dressing led to wound closure in 48% of patients by week 20, surpassing the 30% closure rate in the control group. The trial group exhibited enhanced wound closure compared to the control group by the specified week. Furthermore, the sucrose group demonstrated a higher proportion of wound healing.^35^ In a randomized clinical trial study, dehydrated human amnion/chorion membrane resulted in ulcer healing in 70% of intent‐to‐treat patients with DFUs.^36^ EpiCord and Grafix, as two other similar placenta‐derived products, were reported to have 81% and 82.1% healing in patients receiving these treatments.^37^, ^38^ These novel topical treatments represent some of the most promising therapeutic options available for DFUs, and our current study found that Dermaheal ointment achieved similar results to these treatments in terms of wound healing. Specifically, our study found that complete and partial healing was achieved in 56% and 28% of DFU patients, respectively.

The current study presented herein is the first to investigate the efficacy of Dermaheal topical ointment in treating DFU in human subjects. Before this study, the effectiveness of Dermaheal ointment on cutaneous wounds in diabetic rats was evaluated, and its results were compared to a control group and another experimental group receiving olive leaf extract ointment. The mentioned study demonstrated that Dermaheal ointment significantly improved neutrophil, macrophage, fibroblast, and angiogenesis indices, as well as increased collagen production and organization in the ulcer site, resulting in a significant improvement of diabetic ulcers compared to the control group.^12^ The ingredients of this topical ointment have been studied in the literature before. Dermaheal ointment is formulated based on a combination of natural and herbal products such as *A. euchroma*, *Thymus vulgaris*, *M. chamomilla*, *C. longa*, wax, honey, and olive oil, all of which have anti‐inflammatory, antioxidant, and antimicrobial properties. Therefore, the effectiveness of the ingredients used in this ointment is well‐supported.^16^, ^39^, ^40^ For instance, one recent study examined the topical effects of olive oil and honey to improve DFUs in 45 patients. The group receiving honey and olive oil showed significant improvement in the average tissue score around the wound, the degree of the wound, and the wound healing compared to the control group.^20^

Furthermore, another study that investigated the anti‐inflammatory effects of honey wax, as one of the constituents of Dermaheal, in healing DFUs showed a significant reduction in the wound's size. In this regard, the study of the topical effects of beeswax in treating DFUs also showed a reduction in the size of grade 1 and 2 ulcers.^41^ In our study, the use of Dermaheal ointment in grade 1 and 2 ulcers not only accelerated wound healing but also significantly improved the tissue color, which is an indication of wound improvement. *A. euchroma* is effective for burn wound healing in both human clinical trials and animal model studies.^42^ For instance, in a study on rats with a second‐degree burn, re‐epithelization, fibroblast proliferation, and collagen bundle synthesis were noticeably higher in treatment groups who received *A. euchroma* cream at a concentration of 10% and 20%. The plant's roots contain active constituents, including naphthoquinone derivatives such as alkannin, alkannan, shikonins, and arnebin‐2. These compounds are known for their anti‐inflammatory and wound‐healing properties. Shikonin, a 1,4‐naphthoquinone derivative present in *A. euchroma*, has shown efficacy against methicillin‐resistant *Staphylococcus aureus* and Enterococci. Moreover, it has a historical usage in traditional medicine for the treatment of ulcers and skin diseases.^43^, ^44^ Previous studies suggest that the effectiveness of *A. euchroma* in healing burn wounds may be attributed to its ability to stimulate tissue regeneration through promoting fibroblast proliferation, re‐vascularization, and collagen formation. Furthermore, its antibacterial and anti‐inflammatory properties contribute to its efficacy in the treatment of burn wounds.^43^ Previous research has shown that *M. chamomilla*  contains compounds such as chamazulene, alpha‐bisabolol, bisabolol oxides, spiroethers, and flavonoids. These constituents exhibit anti‐inflammatory, antibacterial, and anti‐fungal properties, as highlighted in earlier studies.^45^ The efficacy of chamomile in wound healing has been substantiated in studies involving both diabetic and nondiabetic rats. For instance, research demonstrated that the topical application of chamomile expedited the healing of burn wounds in rats. This accelerated healing was credited to the stimulation of re‐epithelialization, improved fibroblast function, and increased formation of collagen fibers.^46^ Polyherbal formulations for treating DFU have garnered increased attention from pharmaceutical companies, clinicians, and patients. These formulations are developed based on ingredients previously reported to positively impact the healing process of DFUs, demonstrating non‐inferiority when compared to silver sulfadiazine and standard care treatments (Viswanathan et al., 2011).

In the context of exploring novel treatments, the focus shifts to Dermaheal ointment and its effects on ulcers. Beyond the current study's examination of Dermaheal's impact on ulcers, the ingredients within Dermaheal have been extensively studied in vivo and in vitro, consistently revealing their beneficial effects on DFUs. In essence, the outcomes of this investigation align with cutting‐edge technologies, showcasing Dermaheal's effectiveness in promoting ulcer healing. The thorough examination of these ingredients and their reported efficacy in promoting ulcer healing reinforces the positive impact of Dermaheal ointment. The combination of these carefully studied ingredients within Dermaheal has demonstrated remarkable effectiveness in promoting ulcer healing among diabetic patients, with no significant adverse effects noted. To fortify these findings, conducting further studies with increased statistical power could provide more comprehensive insights into the specific effects of Dermaheal on DFUs.

### 4.1 Study implications

There are various treatments for DFU, including maintaining a moist wound environment. The treatment could be tailored to patients' clinical conditions, preferences, and satisfaction. The use of Dermaheal ointment could improve healing and reduce the size of the DFU, which are preventive factors for deterioration. Topical ointment, as a part of wound care, is pivotal in managing chronic ulcers. Adjunctive and combination therapy have been reported to be more effective than monotherapy in DFU, and it could accelerate wound closure.^47^, ^48^ The utilization of dressings and topical ointments as integral components of wound care is increasingly recognized as crucial in managing DFUs.^4^, ^49^ Topical ointments are easy to use, safe, popular among the population, and effective in treatment. Based on the complex nature of the DFU, one can benefit from administrating topical ointment such as Dermaheal in ulcer management. Expanding on the significance of topical ointments like Dermaheal in DFU management, it is crucial for clinicians to continually assess the evolving landscape of wound care formulations. Regular evaluation of emerging topical agents and advancements in drug delivery systems ensures that the most efficacious and patient‐friendly options are incorporated into treatment regimens. Additionally, conducting targeted clinical trials to evaluate the comparative effectiveness of Dermaheal against other topical interventions can provide evidence‐based insights, guiding clinicians in making informed decisions tailored to individual patient profiles. Moreover, fostering patient education on proper application techniques and the importance of consistent ointment use is essential for maximizing the therapeutic benefits of Dermaheal and similar formulations in promoting DFU healing and preventing complications.

### 4.2 Study limitations

This study had a limited follow‐up time; therefore, further evaluation of refractory DFUs is warranted. Additionally, when interpreting the results, it is essential to acknowledge the potential limitation of patient sampling due to the impact of the Coronavirus Disease 2019 (COVID‐19) pandemic. An additional limitation to consider is the potential variability in patients' adherence to standard care practices, particularly in aspects such as offloading. While the study incorporated standardized protocols for offloading, the extent to which patients followed these recommendations may have varied. Patient compliance with offloading measures, including the use of custom‐made insoles or footwear, can significantly impact DFU healing outcomes. Variations in adherence levels among participants might introduce a source of variability in the results, potentially influencing the observed effects of Dermaheal ointment. Acknowledging and addressing the diversity in patients' engagement with standard care practices, specifically in offloading, would provide a more nuanced understanding of the multifaceted factors influencing DFU management outcomes. Future research might benefit from incorporating measures to assess and account for individual differences in the implementation of standard care components, enhancing the study's internal validity and the broader applicability of its findings.

## 5 CONCLUSION

The use of dressing with Dermaheal ointment, a topical treatment for DFUs composed of several natural ingredients, has shown promise in enhancing the healing process and decreasing the size of DFUs without any observed local or systemic side effects. As such, it can be considered an effective local treatment for DFUs when combined with standard wound care. However, additional research is needed to validate the long‐term effectiveness of these outcomes.

## AUTHOR CONTRIBUTIONS

**Pouya Salahi**: Conceptualization; data curation; methodology; writing—original draft. **Morteza Nasiri**: Data curation; methodology; validation. **Leila Yazdanpanah**: Formal analysis; supervision; validation; visualization; writing—original draft. **Sepehr Khosravi**: Writing—review & editing. **Mohammad Reza Amini**: Conceptualization; funding acquisition; investigation; methodology; project administration; resources; supervision; validation.

## CONFLICT OF INTEREST STATEMENT

The authors declare no conflict of interest.

## ETHICS STATEMENT

This study was approved by the Institutional Review Board and the Ethics Committee of Endocrine and Metabolism Research Institute (EMRI), Tehran University of Medical Sciences (TUMS), Tehran, Iran (No. IR.TUMS.EMRI.REC.1399.036).

## TRANSPARENCY STATEMENT

The lead author Mohammad Reza Amini affirms that this manuscript is an honest, accurate, and transparent account of the study being reported; that no important aspects of the study have been omitted; and that any discrepancies from the study as planned (and, if relevant, registered) have been explained.

## Supporting information

Supporting information.

## Data Availability

The data that support the findings of this study are available on request from the corresponding author.
