# Supplementary material for: Short‐term effect of dressing with Dermaheal ointment in the treatment of diabetic foot ulcer: A double‐blinded randomized controlled clinical trial
Source: Health Sci Rep. 2024 Feb 13;7(2):e1868. doi: 10.1002/hsr2.1868 (PMC10864714; doi:10.1002/hsr2.1868)

**Supplementary Material 1: Diabetic Foot Ulcer Healing Checklist and its Guide**

The “Diabetic Foot Ulcer Healing Checklist” assesses four parameters: ulcer degree, ulcer color, ulcer peripheral tissues, and ulcer exudates. Based on this checklist, each parameter has variables accounting for a part of the maximum 100 score. The total score ranges from 50 (indicative of deterioration) to 400 (indicative of ulcer healing). The method of scoring is as follows:

1. Ulcer degree

Based on the stages, explained below, the total score ranges from 10 to 100.

1. Stages 0, 1, 2, and 3: Score between 80 to 100
2. Stages 3, 4, 5, and 6: Score between 65 to 100
3. Stages 6, 7, 8, and 9: Score between 40 to 65
4. Stages 9, 10, 11, and 12: Score between 25 to 40
5. Stages 12, 13, 14, and 15: Score between 10 to 25
6. Ulcer color
7. Center of ulcer and complete healing (new skin tissue covered the entire area): Score 50
8. Periphery of ulcer and complete healing (new skin tissue covered the entire area): Score 50
9. Red and granulated tissue in the center of the ulcer: Score 40
10. Red and granulated tissue in the periphery of the ulcer: Score 40
11. Yellow tissue in the center of the ulcer: Score 30
12. Yellow tissue in the periphery of the ulcer: Score 30
13. Necrotic tissue in the center of the ulcer: Score 20
14. Necrotic tissue in the periphery of the ulcer: Score 20
15. Necrotic tissue and red granulated tissue in the center of the ulcer: Score 10
16. Necrotic tissue and red granulated tissue in the periphery of the ulcer: Score 10
17. Ulcer peripheral tissue
18. Color

- Normal color: Score 25
- Red color: Score 20
- Pale color: Score 15
- Cyanotic color: Score 10

1. Hotness

- Yes (temperature of the surrounding area of the ulcer is more than core temperature): Score 0
- No (temperature of the surrounding area of the ulcer is not more than core temperature): Score 25

1. Edema

- Yes (skin and surrounding tissue of ulcer is edematous): Score 0
- No (skin and surrounding tissue of ulcer is not edematous): Score 25

1. Sense

- No sensation in the surrounding area of the ulcer: Score 0
- Decreased sensation in the surrounding area of the ulcer: Score 15
- Yes (normal sensation in the surrounding area of the ulcer): Score 25

1. Ulcer exudates
2. Color

- Without exudates: Score 40
- Serosal exudate: Score 30
- Bloody exudate: Score 20
- Yellow exudate: Score 10
- Green exudate: Score 0

1. Odor

- No: Score 20
- Yes: Score 0

1. Amount

- Without exudates: Score 40
- Low (the first layer of dressing is only wet due to contact with exudate): Score 30
- Moderate (exudate is visible in the first layer of dressing but not through the entire dressing layers): Score 20
- Much (visible through the dressing): Score 10

Based on this checklist, the ulcer healing is graded as follows:

1. Total healing: The total score of 400 based on the checklist.
2. Partial healing: The total score of the checklist has at least increased by 30 compared to the baseline score.
3. No healing: The total score of the checklist has increased by less than 30 compared to the baseline score.
4. Deterioration: The total score of the checklist has decreased by at least 10 scores compared to the baseline score.

**Diabetic foot ulcer healing checklist**

|  | **Ulcer Parameters** | **Distribution of scores** | | | | | | | | | | | | | | | | |
| --- | --- | --- | --- | --- | --- | --- | --- | --- | --- | --- | --- | --- | --- | --- | --- | --- | --- | --- |
| **100** | **Ulcer degree** | **Stages** | **0** | **1** | **2** | **3** | **4** | **5** | **6** | **7** | **8** | **9** | **10** | **11** | **12** | **13** | **14** | **15** |
|  |  | **Scores** | **100** | **90** | **90** | **80** | **75** | **65** | **65** | **55** | **50** | **40** | **40** | **30** | **25** | **15** | **15** | **10** |
| **100** | **Ulcer color** | **Center** | **Total healing** | | | **Red (granular)** | | | **Yellow** | | | **Necrotic** | | | **Necrotic + Red (granular)** | | | |
|  |  |  | **50** | | | **40** | | | **30** | | | **20** | | | **10** | | | |
|  |  | **Periphery** | **Total healing** | | | **Red (granular)** | | | **Yellow** | | | **Necrotic** | | | **Necrotic + Red (granular)** | | | |
|  |  |  | **50** | | | **40** | | | **30** | | | **20** | | | **10** | | | |
| **100** | **Ulcer peripheral tissues** | **Color** | **Normal** | | | **Red** | | | **Pale** | | | **Cyanotic** | | | **-** | | | |
|  |  |  | **25** | | | **20** | | | **15** | | | **10** | | | **-** | | | |
|  |  | **Hotness** | **Yes** | | | **No** | | | **-** | | | **-** | | | **-** | | | |
|  |  |  | **0** | | | **25** | | | **-** | | | **-** | | | **-** | | | |
|  |  | **Edema** | **Yes** | | | **No** | | | **-** | | | **-** | | | **-** | | | |
|  |  |  | **0** | | | **25** | | | **-** | | | **-** | | | **-** | | | |
|  |  | **Sense** | **No** | | | **Decreased** | | | **Yes** | | | **-** | | | **-** | | | |
|  |  |  | **0** | | | **15** | | | **25** | | | **-** | | | **-** | | | |
| **100** | **Ulcer exudates** | **Color** | **Without exudates** | | | **Serosal** | | | **Bloody** | | | **Yellow** | | | **Green** | | | |
|  |  |  | **40** | | | **30** | | | **30** | | | **20** | | | **10** | | | |
|  |  | **Odor** | **No** | | | **Yes** | | | **-** | | | **-** | | | **-** | | | |
|  |  |  | **20** | | | **0** | | | **-** | | | **-** | | | **-** | | | |
|  |  | **Amount** | **Without exudates** | | | **Low** | | | **Moderate** | | | **High** | | | **-** | | | |
|  |  |  | **40** | | | **20** | | | **20** | | | **10** | | | **-** | | | |
| **Total score (total healing) = 400 (higher score= better healing)** | | | | | | | | | | | | | | | | | | |

**
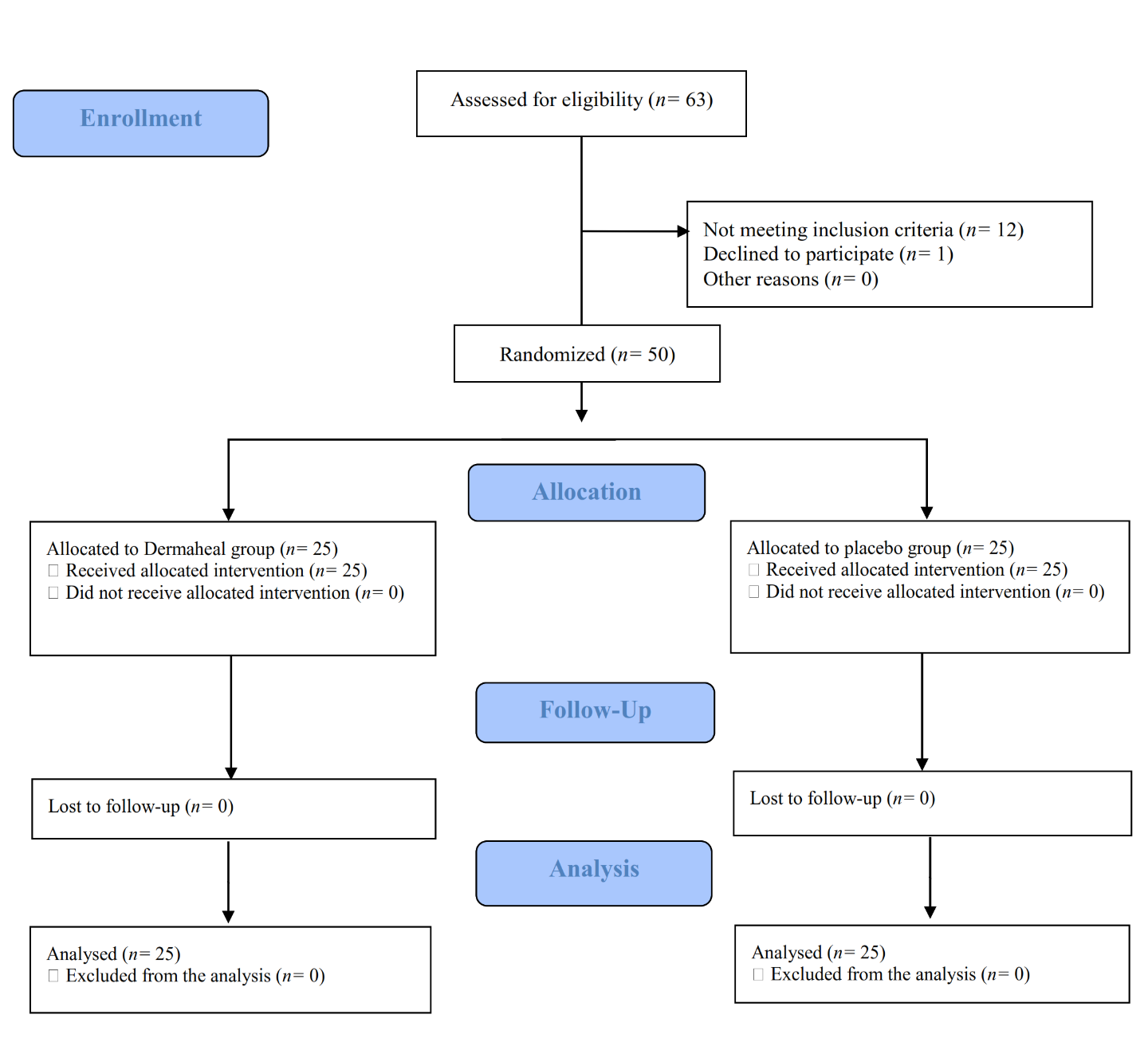
**

**Supplementary Material 2:** The CONSORT flow diagram of the patients’ enrollment, allocation, intervention, follow-up, and analysis

**Supplementary Material 3: Ulcer healing parameters score among patients with diabetic foot ulcer at different times**

| **Parameters**^†^ **(times**^††^**)** | | **Dermaheal group (*n=* 25)** | **Placebo group**  **(*n=* 25)** | **Effect size** | **95% confidence interval** | **Test results**^††^ | | |
| --- | --- | --- | --- | --- | --- | --- | --- | --- |
|  |  |  |  |  |  | **Time** | **Group** | **Time × Group** |
| **Degree** | **Baseline** | 73.2 ± 13.45 | 70.8 ± 16.62 | 0.15 | ˗ 6.20, 11.00 | F= 23.5  P< 0.001 | ‌F= 5.1  P= 0.02 | ‌F= 2.1  P= 0.1 |
|  | **1^st^ week** | 80.6 ± 9.05 | 72.6 ± 13.92 | 0.68 | 1.32, 14.67 |  |  |  |
|  | **2^nd^ week** | 82.5 ± 10.32 | 74.3 ± 14.84 | 0.63 | 0.69, 15.54 |  |  |  |
|  | **3^rd^ week** | 85.9 ± 10.98 ^a,b^ | 77.3 ± 12.95 | 0.70 | 1.28, 15.75 |  |  |  |
|  | **4^th^ week** | 91.5 ± 6.5 ^a,b,c,d^ | 79.5 ± 12.99 ^c^ | 1.16 | 5.44, 18.46 |  |  |  |
| **Color** | **Baseline** | 68.8 ± 17.86 | 68.4 ± 14.62 | 0.02 | ˗ 8.88, 9.68 | F= 12.7  P< 0.001 | ‌F= 3.9  P= 0.05 | ‌F= 5.0  P= 0.006 |
|  | **1^st^ week** | 76.4 ± 16.29 ^a^ | 66.8 ± 17.25 | 0.57 | 0.05, 19.14 |  |  |  |
|  | **2^nd^ week** | 79.1 ± 15.58 ^a^ | 70.0 ± 19.78 | 0.51 | ˗ 1.17, 19.51 |  |  |  |
|  | **3^rd^ week** | 83.6 ± 19.15 ^a^ | 70.4 ± 22.14 | 0.63 | 0.58, 25.78 |  |  |  |
|  | **4^th^ week** | 92.5 ± 11.18 ^a,b,c,d^ | 73.3 ± 24.15 | 1.01 | 7.17, 31.15 |  |  |  |
| **Peripheral tissues** | **Baseline** | 66.2 ± 19.54 | 65.0 ± 26.73 | 0.05 | ˗ 12.11, 14.51 | F= 7.9  P< 0.001 | F= 2.0  P= 0.1 | F= 0.09  P= 0.9 |
|  | **1^st^ week** | 75.0 ± 17.73 | 64.4 ± 24.25 | 0.49 | ˗ 1.48, 22.68 |  |  |  |
|  | **2^nd^ week** | 76.8 ± 19.71 | 68.1 ± 21.40 | 0.42 | ˗ 3.20, 20.70 |  |  |  |
|  | **3^rd^ week** | 79.5 ± 16.02 | 71.5 ± 18.66 | 0.45 | ˗ 2.63, 18.54 |  |  |  |
|  | **4^th^ week** | 81.7 ± 8.47 ^a^ | 73.8 ± 18.43 ^c^ | 0.55 | ˗ 1.20, 17.08 |  |  |  |
| **Exudates** | **Baseline** | 85.2 ± 20.23 | 82.0 ± 21.60 | 0.15 | ˗ 8.70, 15.10 | F= 13.6  P< 0.001 | ‌F= 1.3  P= 0.2 | F= 0.3  P= 0.7 |
|  | **1^st^ week** | 90.8 ± 14.97 | 88.8 ± 16.66 | 0.12 | ˗ 7.00, 11.00 |  |  |  |
|  | **2^nd^ week** | 93.3 ± 11.29 | 87.5 ± 17.25 | 0.39 | ˗ 2.64, 14.30 |  |  |  |
|  | **3^rd^ week** | 97.2 ± 7.02 | 91.3 ± 13.9 | 0.53 | ˗ 0.79, 12.61 |  |  |  |
|  | **4^th^ week** | 100.0 ± 0.001 ^a^ | 94.2 ± 11.21 ^a^ | 0.72 | 0.64, 10.78 |  |  |  |
| *Note:* All values are reported as means ± standard deviations.  ^†^ Diabetic foot ulcer healing was measured by a four-parameter scale: each parameter obtains a maximum score of 100.  ^††^ The outcomes were recorded at five points of time, including before the intervention (baseline), and on the first, second, third, and fourth weeks of intervention.  ^††^ Repeated-measures analysis of variance (Greenhouse-Geisser).  ^a^ Significant compared to the baseline.  ^b^ Significant compared to the second week.  ^c^ Significant compared to the first week.  ^d^ Significant compared to the third week. | | | | | | | | |

**Supplementary Material 4: Changes in ulcer healing parameters among patients with diabetic foot ulcer compared to the baseline on the first, second, third, and fourth weeks of the intervention**

| **Parameters**^†^ **(times**^††^**)** | | **Dermaheal group (*n=* 25)** | **Placebo group**  **(*n=* 25)** | **Effect size** | **95% confidence interval** | **Test results**^††^ |
| --- | --- | --- | --- | --- | --- | --- |
| **Degree** | **1^st^ week** | 7.9 ± 1.62 | 1.2 ± 1.62 | 0.81 | 2.00, 11.26 | F= 31.2, P< 0.001 |
|  | **2^nd^ week** | 10.4 ± 2.20 | 3.9 ± 2.20 | 0.59 | 0.59, 13.39 | F= 4.8, P= 0.03 |
|  | **3^rd^ week** | 14.0 ± 2.26 | 6.9 ± 2.21 | 0.66 | 0.69, 13.52 | F= 5.0, P= 0.03 |
|  | **4^th^ week** | 19.8 ± 2.19 | 8.5 ± 2.08 | 1.05 | 5.10, 17.37 | F= 13.7, P= 0.01 |
| **Color** | **1^st^ week** | 7.6 ± 2.89 | ˗ 1.6 ± 2.89 | 0.64 | 1.15, 17.61 | F= 5.2, P= 0.02 |
|  | **2^nd^ week** | 10.5 ± 3.25 | 1.5 ± 3.25 | 0.55 | ˗ 0.29, 18.21 | F= 3.7, P= 0.05 |
|  | **3^rd^ week** | 16.1 ± 3.99 | 2.4 ± 3.99 | 0.58 | 2.26, 25.08 | F= 5.8, P= 0.02 |
|  | **4^th^ week** | 25.4 ± 4.07 | 5.7 ± 3.98 | 0.97 | 8.16, 31.25 | F= 11.9, P= 0.001 |
| **Peripheral tissues** | **1^st^ week** | 9.0 ± 3.21 | ˗ 0.8 ± 3.22 | 0.61 | 0.73, 19.01 | F= 4.7, P= 0.03 |
|  | **2^nd^ week** | 12.4 ± 3.63 | 4.2 ± 3.63 | 0.44 | ˗ 2.19, 18.52 | F= 2.5, P= 0.1 |
|  | **3^rd^ week** | 13.6 ± 3.23 | 8.1 ± 3.23 | 0.33 | ˗ 3.82, 14.76 | F= 1.4, P= 0.2 |
|  | **4^th^ week** | 15.8 ± 2.85 | 10.1 ± 2.78 | 0.40 | ˗ 2.34, 13.87 | F= 2.0, P= 0.1 |
| **Exudates** | **1^st^ week** | 6.3 ± 2.2 | 6.0 ± 2.22 | 0.02 | ˗ 6.08, 6.60 | F= 0.0, P= 0.9 |
|  | **2^nd^ week** | 9.7 ± 2.45 | 5.2 ± 2.45 | 0.36 | ˗ 2.50, 11.50 | F= 1.6, P= 0.2 |
|  | **3^rd^ week** | 14.6 ± 2.15 | 9.8 ± 2.15 | 0.44 | ˗ 1.36, 10.98 | F= 2.4, P= 0.1 |
|  | **4^th^ week** | 16.3 ± 1.62 | 11.1 ± 1.58 | 0.64 | 0.54, 9.77 | F= 5.1, P= 0.02 |
| *Note:* All values are reported as means ± standard errors.  ^†^ Diabetic foot ulcer healing was measured by a four-parameter scale: each parameter obtains a maximum score of 100.  ^††^ The outcomes were recorded at five points of time, including before the intervention (baseline), and on the first, second, third, and fourth weeks of intervention.  ^††^ Analysis of covariance, considering baseline values as covariates. | | | | | | |

**Supplementary Material 5:** **The ulcer healing process in one patient of the Dermaheal group during the four weeks of intervention**


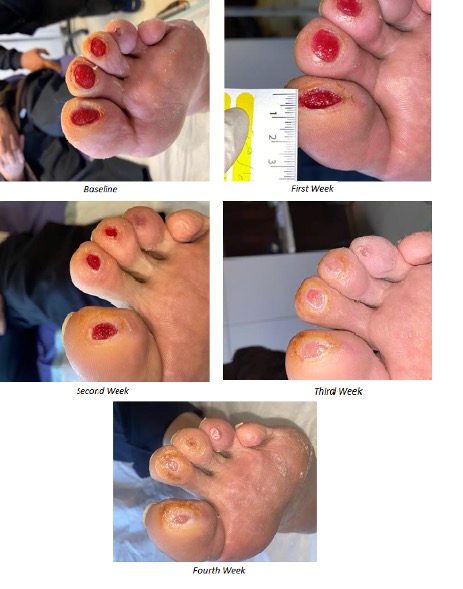

Supplement: Supplementary file 1 — Supporting information. [file HSR2-7-e1868-s001.docx]
